# A comparison of Irish set dancing and exercises for people with Parkinson’s disease: A phase II feasibility study

**DOI:** 10.1186/1471-2318-13-54

**Published:** 2013-06-04

**Authors:** Daniele Volpe, Matteo Signorini, Anna Marchetto, Timothy Lynch, Meg E Morris

**Affiliations:** 1 Department of Physical Medicine & Rehabilitation, S. Raffaele Arcangelo Fatebenefratelli Hospital, Cannaregio, Venice, 3458, Italy; 2 Fatebenefratelli Association for Research, Rome, Italy; 3 Neurological Institute, Mater Misericordiae University Hospital, Dublin, Ireland; 4 Department of Physiotherapy, School of Allied Health, La Trobe University, La Trobe, 3086, Australia

**Keywords:** Parkinson’s disease, Dancing, Rehabilitation, Exercise therapy, Balance, Randomized controlled trial, Geriatrics

## Abstract

**Background:**

People with idiopathic Parkinson’s disease (PD) frequently have low activity levels, poor mobility and reduced quality of life. Although increased physical activity may improve mobility, balance and wellbeing, adherence to exercises and activity programs over the longer term can be challenging, particularly for older people with progressive neurological conditions such as PD. Physical activities that are engaging and enjoyable, such as dancing, might enhance adherence over the long term. The objective of this study was to evaluate the feasibility of a randomized controlled trial of Irish set dancing compared with routine physiotherapy for people with mild to moderately severe PD.

**Methods:**

Twenty-four people with idiopathic PD referred for movement rehabilitation were randomized to receive standard physiotherapy exercises or Irish set dancing classes once per week plus a weekly home program for 6 months (12 in each group). The feasibility and safety of the proposed RCT protocol was the main focus of this evaluation. The primary outcome was motor disability measured by the motor component of the UPDRS, which was assessed prior to and after therapy by trained assessors blinded to group assignment. The Timed Up and Go, the Berg Balance Scale and the modified Freezing of Gait Questionnaire were secondary measures. Quality of life of the people with PD was evaluated using the PDQ-39.

**Results:**

Both the Irish set dancing and physiotherapy exercise program were shown to be feasible and safe. There were no differences between groups in the rate of adverse events such as falls, serious injuries, death or rates of admission to hospital. The physiotherapists who provided usual care remained blind to group allocation, with no change in their standard clinical practice. Compliance and adherence to both the exercise and dance programs were very high and attrition rates were low over the 6 months of therapy. Although improvements were made in both groups, the dance group showed superior results to standard physiotherapy in relation to freezing of gait, balance and motor disability.

**Conclusions:**

Irish dancing and physiotherapy were both safe and feasible in this sample from Venice, with good adherence over a comparatively long time period of 6 months. A larger multi-centre trial is now warranted to establish whether Irish set dancing is more effective than routine physiotherapy for enhancing mobility, balance and quality of life in people living with idiopathic PD.

**Trial registration:**

EudraCT number 2012-005769-11

## Background

Idiopathic Parkinson’s disease (PD) is a progressive neurological condition associated with reduced physical activity, poor mobility, falls and reduced health related quality of life. Although there is growing evidence showing that physical activity and exercise programs for people with PD can improve strength [1], balance [2,3], mobility [4] and life quality [5,6], many trials have shown limited long term benefits despite short term gains [7-9]. In addition, adherence to exercises and physical activity programs over the longer term can be problematic in people with PD, particularly for those who are elderly, have advanced disease or for those who have several co-morbidities [7]. Reduced physical activity and poor mobility can be associated with an increased risk of falls, immobility, hospitalization and the need for long term care [6,10-13]. To optimize health related quality of life there is a need to find effective, enjoyable and sustainable methods of exercising in people with this debilitating and progressive disease [14,15].

Dancing is a popular mode of physical activity for many older people. Preliminary trials in PD by Duncan and Earhart [16], Foster [17], Marchant et al. [18] and Hackney and colleagues [19] have shown that dancing can be associated with improvements in mobility, balance and quality of life. It is not clear whether dancing is more effective, sustainable and enjoyable over the long term compared to conventional therapeutic exercises. It is also not known which dancing genre is most effective (such as Irish set dancing, tango, square dancing, ballroom dancing, creative dancing, or improvisation). Whether dancing should be partnered or un-partnered, what music is most suitable and the most effective dosage of dancing to achieve therapeutic benefits remain to be established. Importantly, it is not known if dancing is safe for frail people with PD who have a propensity to fall.

As a prelude to a large scale multi-centre randomized controlled trial (RCT), there is a need to explore the effects of Irish set dancing for people living with PD. Irish set dancing is popular for people with PD throughout the world. It provides rhythmical auditory cues, affords the stability provided by partners, is relatively simple and easy to learn and perform and is reported to be enjoyable [16].

Whilst there is some evidence showing tango dancing to be beneficial for some individuals with PD [19], this form of dance is comparatively complex and challenging and possibly is most effective for people with mild to moderately severe PD. There is a need to test the feasibility, safety and adherence to a long term program of Irish set dancing compared with standard physiotherapy exercises.

Safety is particularly important in people with PD, given the prevalence of balance impairment and falls. Hiorth et al. [20] showed that up to 60% of people with PD fall over a 12 month period. Our feasibility trial will enable us to evaluate which measurement tools best quantify physical activity, balance and mobility over a 6 month period. Furthermore it will allow us to examine whether increasing physical activity levels using Irish set dancing delivered in a rehabilitation setting is associated with contamination in the standard exercise group, leading to changes in usual care activities.

Thus in this feasibility study we address the following research questions:

1. Is the proposed RCT protocol comparing Irish set dancing with routine physiotherapy exercises feasible and safe?

2. Which measures of mobility, balance and quality of life are feasible for people with mild to moderate PD undergoing a 6 month period of movement rehabilitation incorporating dance?

3. What are the estimates of the treatment effects and the estimates of the variance of the treatment effects, as the basis for estimating the sample size needed in a future large scale RCT to ascertain if Irish set dancing is more effective than routine exercises for people with mild to moderately severe PD?

## Methods

### Design

We conducted a single blind, parallel group RCT with a focus on feasibility as a primary outcome. People with idiopathic PD were recruited from the Parkinson’s disease Association in Venice, Italy. The trial was approved by the hospital ethics committee (C.E.O.C. Brescia Italy, ref 326/2012). The trial was registered with the EudraCT (2012-005769-11). Written informed consent was obtained from the participants who scored more than 24/30 on the Mini Mental State Examination. If appropriate, written informed consent was obtained from the spouses of participants who scored less than 25/30. Participants were randomized to receive a program of either Irish set dancing or standard physiotherapy exercises for 1.5 hours once per week for 6 months.

### Participants

Participants were eligible for inclusion if they had idiopathic Parkinson’s disease as diagnosed by a medical practitioner and were rated level 0–2.5 on the modified Hoehn & Yahr scale [21]. We included only those with mild to moderately severe PD for safety reasons as people at stage 3 or more on the Hoehn and Yahr scale have a high risk of falls. Participants were excluded if they did not speak Italian, if they had co-morbidities that prevented dancing, mobility or safe exercise, if they had received deep brain stimulation surgery or if they were unable to travel to the dancing or physiotherapy venues.

### Randomization

After initial screening procedures and baseline testing, we used a blocked stratified randomization procedure, based on the modified Hoehn & Yahr (1967) score, to allocate participants to one of the two groups. We stratified according to modified Hoehn & Yahr (1967) scale scores to ensure that groups had similar proportions of those who were mild (H&Y 0–1.5) and moderately affected (2–2.5). We used computer generated number sequences for randomization and this procedure was conducted by a third party. Opaque envelopes were used to conceal allocation. Trained assessors who were blinded to group allocation conducted all of the assessments. Physiotherapists and dancing teachers providing the intervention could not be blinded to group allocation. To reduce the risk of contamination by the usual care staff becoming aware of group assignment, we therefore ensured that employers not directly involved in the study were not told of the aims, hypotheses or predictions of the trial. The therapists and dancing teachers who provided the interventions were not involved in other aspects of patient care. To test whether staff remained blind to group allocation, we asked them to guess group allocation at the end of the trial. Seven of the 24 subjects guessed their group assignment correctly.

### Intervention

Participants in the Irish set dancing group received a 90 minute set dancing class weekly for six months in a dance studio located in Venice. The dance classes were held by two set dancing teachers of the Blacksheep Irish Set Dancing School in Venice, Italy. People with PD were partnered with members of the Irish set dancing school to ensure their safety, as advised by Earhart [22]. Family members were also invited to assist or partner the people with PD. The Irish set dancing class included a preliminary warm up consisting of range of movement, balance and postural exercises [23]. The goal of the warm up was to prepare people with PD for the set dancing class focusing on steps, turning, balance and posture. The class used different set dancing steps and in particular reel and polka steps. The teachers taught sets, in group formation, in four pairs of two. They were taught sets from different counties of Ireland and in particular the Corofin plain reel set, the Antrim reel set, the Black Valley Jig square, and the Durrow Threshing polka set. The sets were chosen to improve motor symptoms in Parkinson’s disease such as balance impairment, freezing of gait and hypokinesia using Irish music as a rhythmical cue [14]. Each class ended with a group dance in a circle and then with relaxation exercises. The protocol incorporated 10 minutes of warm up range of movement, balance and postural exercises, 70 minutes of Irish dance lessons and a 10 minutes cool down. Each person with PD was also given a video with recordings of the steps danced by the teacher. They were requested to watch the video at home once during each week, for a period of 1 hour.

The weekly standard physiotherapy exercise sessions included individual sessions delivered by a physiotherapist or physiotherapy assistant designed to improve muscle strength, mobility, balance, and postural control. The physiotherapy program was in according to the KNGF Guidelines for physical therapy in Parkinson's disease as described below. For each session each person had warm up range of movement and stretching exercises for 10 minutes followed by 50 minutes of strength training, balance training and postural re- education, then 20 minutes of gait training and a 10 minute cool down.

**Table 1:** Demographic and clinical data

**Variable**	**PD Irish dance (n = 12)**	**PD Physiotherapy (n = 12)**
mean ± standard deviation (range)		
Gender (M/F)	7/5	6/6
Age (yr)	61.6 ± 4.5 (58 – 73)	65.0 ± 5.3 (56 – 71)
Height (cm)	167.6 ± 6.4 (157 – 180)	168.6 ± 6.6 (159 – 183)
Weight (Kg)	71.3 ± 6.0 (65 – 87)	70.3 ± 6.8 (60 – 84)
Disease duration (yr)	9.0 ± 3.6 (5 – 18)	8.9 ± 2.5 (5 – 13)
Hoehn-Yahr score	2.2 ± 0.4 (2 – 3)	2.2 ± 0.4 (2–3)
MMSE score	26.5 ± 1.4 (24 – 29)	26.3 ± 1.8 (24 – 29)
Levodopa (mg/day)(n = 24)	725.0 ± 234 (400 – 1000)	645.0 ± 216 (400–1000)
*Dopamine agonist (mg/day)*		
Pramipexole E.R.	(n = 5) 1.9 ± 0.5 (1.05 – 2.1)	(n = 4) 2.4 ± 0.5 (2.1–3.15)
Ropinirole E.R.	(n = 4) 12.5 ± 1.9 (10 – 14)	(n = 5)12.0 ± 2 (10–14)
Rotigotine	(n = 3) 12.0 ± 2 (10–14)	(n = 4)13.5 ± 2.5 (10–16)
Rasagiline	(n = 1) 1	(n = 3) 1
*Other drugs (mg/day)*		
Entacapone	(n = 3) 1000±200 (800 – 1200)	(n = 2) 1000

Each person with PD was given a video with recordings of the physiotherapy exercises. They were requested to watch the video at home once during each week, for a period of 1 hour. Over the six month intervention period, participants had on average 21.08 individual sessions of physiotherapy and 21.83 dance classes.

### Details of program content

#### Standard physiotherapy program according to the KNGF guidelines for physical therapy in Parkinson’s disease

•Cognitive movement strategies:*to divide complex automatic activities into simple movements avoiding dual tasking and to practice the movements and rehearsed in the mind.*

•Cueing strategies: *to improve movement and gait impairments with cueing (auditory, visual and proprioceptive cueing)*

•Improvement of transfers: *to train transfers by applying cognitive movement strategies and cues to initiate and continue movement*

•Normalizing body posture: *to preventing or treating postural deformities with exercises for postural realignment and coordinated movements*

•Training reaching and grasping: *to improve reaching and grasping, and manipulation of objects using cueing strategies and cognitive movement strategies avoiding dual tasking.*

•Training balance: *to optimize balance during the performance of activities with exercises for balance and training strength. Falls prevention strategies*

•Gait training: *to walk safely and to increase (comfortable) walking speed with the use of cues and cognitive movement strategies and to train musclestrength and mobility of the trunk and upper and lower limbs.*

•Improvement of physical capacity: *to maintain or to improve physical capacity with training of aerobic muscle strength (with the emphasis on the muscles of the trunk and legs), joint mobility (among others, axial) and muscle length (among others, muscles of the calf and hamstrings)*

### Outcome measures

We assessed outcomes at two time points. Baseline measures were taken within 3 weeks prior to therapy. The second assessment occurred within 3 weeks of the final week of the six month therapy period. The final assessment was 3 weeks after discharge. These time points were chosen because we wanted to evaluate the attainment and retention of skills learned during the Irish dancing course or physiotherapy classes [24]).

So we could compare our findings with other trials on rehabilitation outcomes in PD, we measured motor disability using the motor component of the UPDRS [25]. We also tested participants using the Timed Up and Go, the Berg Balance Scale and the modified Freezing of Gait Questionnaire as secondary measures of outcome. We also quantified health related quality of life in all participants using the PDQ-39. Demographic data collected at baseline included age, sex, duration PD, PD medications, cognition assessed using the Mini Mental State Examination. Although these tools have been tested for reliability and validity in other clinical trials, we wanted to examine their clinic-metric properties in relationship to a dance program over a 6 month period.

#### Adverse events

We recorded all adverse events such as injuries, distress, falls, deaths, and hospital admissions as a results of injures from the programs.

### Statistical analysis

This clinical trial used a sample of convenience, with the assumption that 24 participants would be ample to explore safety and feasibility. For each group we compared for each variable changes in performance from admission to discharge. We also evaluated group by time interactions using repeated measures analysis of variance. We also report the effect size estimates for the treatment effects and the variance in the treatment effects. The amount of time spent in each type of therapy was recorded by a physiotherapist or therapy assistant. In addition, at the beginning of each session, participants were required to sign a form in order to attest their attendance. Data on hospital admission rates, injuries and adverse events were verified by phone interview. There were no deaths during the trial.

## Results

### Participants

Table 1 shows the baseline characteristics for both groups to be comparable. There were no statistically significant differences between groups, including for key variables such as height, weight, PD duration, H-Y or MMSE scores.

#### Intervention dosages

For the sample as a whole, 89.4% of intervention sessions were delivered. Only 10.6% of sessions were not delivered due to personal reasons or due to illnesses not related to Parkinson’s disease. More specifically, 90.9% of Irish dance classes and 87.8% physiotherapy sessions were provided.

#### Blinding & contamination

The trained assessors, blinded to group assignment, were unable to identify to which group participants were assigned (29% of the group assignments were guessed correctly). There was no significant difference in the time spent in therapy between the standard physiotherapy group (mean 1898 minutes) and Irish set dancing group (mean 1965 minutes).

#### Therapy outcomes

Table 2 provides the means and standard deviations at admission and at discharge for each of the key variables. The table shows that the Irish dancing group made greater gains than the standard physiotherapy group. Analysis of variance for the UPDRS III motor section scores showed better results for the Irish dancing group (F (1, 23) = 6.35, p =.019). Paired t-tests showed improvements from admission to discharge in both the physiotherapy group (t (11) = 5.841, p<.001) and the Irish dance group: (t (11) =12.46, p<.001).

**Table 2:** Means and (standard deviations) for key variables for each group before and after intervention

	**Irish dancing**	**Physiotherapy**
Motor UPDRS Baseline	24.58 (3.87)	23.92 (3.50)
Motor UPDRS Discharge	17.42 (3.85)	21.00 (3.07)
Berg Balance Scale Baseline	36.08 (9.20)	34.08 (9.14)
Berg Balance Scale Discharge	46.08 (6.75)	38.92 (9.97)
FOG Baseline	11.42 (2.78)	10.75 (3.39)
FOG Discharge	4.92 (2.07)	10.16 (4.47)
PDQ39 Baseline	30.60 (12.06)	32.58 (7.59)
PDQ39 Discharge	22.16 (10.18)	27.61 (7.67)

Analysis of variance for the TUG test showed superior results over time for the Irish dancing group (F (1, 23) = 8.938, p =.007). Paired t-tests showed improvements from admission to discharge in both the physiotherapy group (t (11) = 5.841, p <.001) and the Irish dance group: (t (11) = 9.666, p<.001).

For the Berg Balance Scale a trend towards similar results was seen. The Irish set dance group made gains (F (1 ,23) = 4.254; p =.051) although this was not significantly different from standard therapy.

For the FOG questionnaire the Irish set dancing group made significant gains t (df = 11) =16.296, p =.000). Analysis of variance also showed the group by phase interaction to be statistically significant (F (1, 23) =13.648, p = .001).

For the PDQ-39 the Irish set dance group and the physiotherapy group showed similar outcomes (F (1, 23) = 2.192, p =.153).

#### Adverse events

Over the six months treatment period 19 participants experienced a non-injurious fall (10 control group, 9 Irish dance group). Only one fall occurred during the Irish set dancing, and no falls occurred during physiotherapy exercise classes. The fall was not injurious and no medical attention was sought. None of the participants were admitted to hospital during their rehabilitation.

#### Sample size estimations for future trials

We used the discharge standard deviations and estimates of clinically significant minimal effects sizes to calculate sample sizes for a future large trial. We aimed to detect an effect size of 0.8, allowing for 20% dropouts, based on the assumption of two groups of equal size and a two tailed significance threshold alpha of 0.05. For the UPDRS motor, is estimated that 18 people are required per group. For the Berg balance scale, it is estimated that 67 people are required per group. For the FOG test it is estimated that 9 people are required per group. Finally, for the PDQ 39 total score, it is estimated that 52 people are required per group. Thus we recommend 70 per group for a future trial, allowing for small levels of non-compliance.

## Discussion

We have shown the feasibility of a RCT of Irish set dancing compared to standard physiotherapy for people with idiopathic Parkinson’s disease. Both of the programs were delivered successfully over a 6 month time period with very good attendance and good adherence. People with Parkinson’s disease appeared to have the potential to benefit from dancing classes that incorporate elements such as rhythmical music, large amplitude fast movements, dancing with partners and step routines. What is not known is whether other dance genres, such as ballroom dancing, Tango, modern dance, square dancing, jazz ballet or classical ballet are more effective at improving movement, balance and quality of life in people with PD. Moreover the therapeutic effects of the different elements of Irish dancing, such as reel, polka and jig steps have not been quantified. The study did not compare dancing genres or the content and duration of dancing. The extent to which different types of music contribute to outcomes also remains open to question.

In terms of outcome measures, we were able to quantify motor disability using the motor component of the UPDRS, the Timed Up and Go, the Berg Balance Scale and the modified Freezing of Gait Questionnaire. Of these the UPDRS was best able to discriminate change and was quick and easy to administer. We also found it feasible to measure life quality using the PDQ-39.

There were a small number of limitations of this pilot study. Firstly, PD medications were not controlled, simply documented and testing did not always occur at peak dose in the medication cycle despite being always in the same hour. In addition the content of the standard physiotherapy treatment program reflected clinical practice in Italy and may not necessarily reflect contemporary clinical practice in other parts of the world even if the physiotherapy program was in according to the KNGF Guidelines for physical therapy in Parkinson's disease. The clinical relevance also needs to be considered given the modest effect sizes reported in this trial. Although modest changes were seen, this is argued to be clinically significant given that Parkinson’s disease is a progressive and debilitating disease for which any benefits to mobility and quality of life can be considered to be helpful.

## Conclusion

To conclude, Irish set dancing appears to be safe, simple, and enjoyable with the potential to improve mobility, reduce disability and enhance health related quality of life. This preliminary investigation provides data showing our protocol for a large scale RCT to be both safe and feasible. The large trial is now needed to ascertain the effectiveness of Irish set dancing compared with routine physiotherapy exercises over the long term.

## Competing interests

The authors declare they have no competing interests.

## Authors’ contributions

DV conceived the study, participated in the design, oversaw data collection, contributed to data interpretation and drafted the manuscript, was responsible for statistical analysis and analyzing the results and contributed to manuscript preparation. MS and AM contributed to statistical analysis and interpretation of data. TL made substantial contribution to conception and design of the study. MEM contributed to data checking and writing the manuscript. All authors read and approved the final manuscript.

## Pre-publication history

The pre-publication history for this paper can be accessed here:

http://www.biomedcentral.com/1471-2318/13/54/prepub

## References

[B1] Li F Harmer P Fitzgerald K Eckstrom E Stock R Galver J Maddalozzo G Batya SS Tai chi and postural stability in patients with Parkinson's disease N Engl J Med 2012 366 6 511 519 10.1056/NEJMoa1107911 22316445 PMC3285459

[B2] Hirsch MA Toole T Maitland CG Rider RA The effects of balance training and high- intensity resistance training on persons with idiopathic Parkinson's disease Arch Phys Med Rehabil 2003 84 8 1109 1117 10.1016/S0003-9993(03)00046-7 12917847

[B3] Allen NE Sherrington C Paul SS Canning CG Balance and falls in Parkinson's disease: a meta-analysis of the effect of exercise and motor training Mov Disord 2011 26 9 1605 1615 10.1002/mds.23790 21674624

[B4] Ashburn A Fazakarley L Ballinger C Pickering R McLellan LD Fitton C A randomised controlled trial of a home based exercise programme to reduce the risk of falling among people with Parkinson's disease J Neurol Neurosurg Psychiatry 2007 78 7 678 684 17119004 10.1136/jnnp.2006.099333 PMC2117667

[B5] Goodwin VA Richards SH Henley W Ewings P Taylor AH Campbell JL An exercise intervention to prevent falls in people with Parkinson's disease: a pragmatic randomised controlled trial J Neurol Neurosurg Psychiatry 2011 82 11 1232 1238 10.1136/jnnp-2011-300919 21856692

[B6] Soh SE Morris ME McGinley JL Determinants of health-related quality of life in Parkinson's disease: a systematic review Parkinsonism Relat Disord 2011 17 1 1 9 10.1016/j.parkreldis.2010.08.012 20833572

[B7] Morris ME Iansek R Kirkwood B A randomized controlled trial of movement strategies compared with exercise for people with Parkinson's disease Mov Disord 2009 24 1 64 71 10.1002/mds.22295 18942100

[B8] Munneke M Nijkrake MJ Keus SH Kwakkel G Berendse HW Roos RA Borm GF Adang EM Overeem S Bloem BR Efficacy of community-based physiotherapy networks for patients with Parkinson's disease: a cluster-randomised trial Lancet Neurol 2010 9 1 46 54 10.1016/S1474-4422(09)70327-8 19959398

[B9] Rochester L Rafferty D Dotchin C Msuya O Minde V Walker RW The effect of cueing therapy on single and dual-task gait in a drug naive population of people with Parkinson's disease in northern Tanzania Mov Disord 2010 25 7 906 911 10.1002/mds.22978 20175212

[B10] Lamont RM Morris ME Woollacott MH Brauer SG Community walking in people with Parkinson's disease Parkinson's disease 2012 2012 856237 22191078 10.1155/2012/856237 PMC3236447

[B11] McGinley JL Martin C Huxham FE Menz HB Danoudis M Murphy AT Watts JJ Iansek R Morris ME Feasibility, safety, and compliance in a randomized controlled trial of physical therapy for Parkinson's disease Parkinsons Dis 2012 2012 795294 22191076 10.1155/2012/795294 PMC3236432

[B12] Tan D Danoudis M McGinley J Morris ME Relationships between motor aspects of gait impairments and activity limitations in people with Parkinson's disease: a systematic review Parkinsonism Relat Disord 2012 18 2 117 124 10.1016/j.parkreldis.2011.07.014 22093237

[B13] Cameron D Murphy A Morris ME Raghav S Iansek R Planned stopping in people with Parkinson Parkinsonism Relat Disord 2010 16 3 191 196 10.1016/j.parkreldis.2009.11.008 20005146

[B14] Morris ME Martin CL Schenkman ML Striding out with Parkinson disease: evidence- based physical therapy for gait disorders Phys Ther 2010 90 2 280 288 10.2522/ptj.20090091 20022998 PMC2816030

[B15] Morris ME Locomotor training in people with Parkinson disease Phys Ther 2006 86 10 1426 1435 10.2522/ptj.20050277 17012646

[B16] Duncan RP Earhart GM Randomized controlled trial of community-based dancing to modify disease progression in Parkinson disease Neurorehabil Neural Repair 2012 26 2 132 143 10.1177/1545968311421614 21959675

[B17] Foster ER Golden L Duncan RP 2012 Earhart GM: A community-based Argentine tango dance program is associated with increased activity participation among individuals with Parkinson disease. Arch Phys Med Rehabil 10.1016/j.apmr.2012.07.028 PMC3557593 22902795

[B18] Marchant D Sylvester JL Earhart GM Effects of a short duration, high dose contact improvisation dance workshop on Parkinson disease: a pilot study Complement Ther Med 2010 18 5 184 190 10.1016/j.ctim.2010.07.004 21056841

[B19] Hackney ME Earhart GM Short duration, intensive tango dancing for Parkinson disease: an uncontrolled pilot study Complementary therapies in medicine 2009 17 4 203 207 10.1016/j.ctim.2008.10.005 19632547 PMC2731655

[B20] Hiorth YH Lode K Larsen JP Frequencies of falls and associated features at different stages of Parkinson's disease European journal of neurology: the official journal of the European Federation of Neurological Societies 2012 10.1111/j.1468-1331.2012.03821.x 22816560

[B21] Hoehn MM Yahr MD Parkinsonism: onset, progression and mortality Neurology 1967 17 5 427 442 10.1212/WNL.17.5.427 6067254

[B22] Earhart GM Ellis T Nieuwboer A Dibble LE Rehabilitation and Parkinson's disease Parkinsons Dis 2012 2012 371406 22550611 10.1155/2012/371406 PMC3328152

[B23] Heiberger L Maurer C Amtage F Mendez-Balbuena I Schulte-Monting J Hepp-Reymond MC Kristeva R Impact of a weekly dance class on the functional mobility and on the quality of life of individuals with Parkinson's disease Front Aging Neurosci 2011 3 14 22013420 10.3389/fnagi.2011.00014 PMC3189543

[B24] Nieuwboer A Rochester L Muncks L Swinnen SP Motor learning in Parkinson's disease: limitations and potential for rehabilitation Parkinsonism Relat Disord 2009 15 Suppl 3 S53 58 20083008 10.1016/S1353-8020(09)70781-3

[B25] Metman LV Myre B Verwey N Hassin-Baer S Arzbaecher J Sierens D Bakay R Test- retest reliability of UPDRS-III, dyskinesia scales, and timed motor tests in patients with advanced Parkinson's disease: an argument against multiple baseline assessments Mov Disord 2004 19 9 1079 1084 10.1002/mds.20101 15372601

